# Case Report: Low-frequency tibial nerve stimulation: demonstrating a novel therapeutic option for Fowler’s syndrome through a pilot case series

**DOI:** 10.3389/fruro.2026.1854503

**Published:** 2026-05-20

**Authors:** Aidan McConnell-Trevillion, Ingrid Hoeritzauer, Helen Simpson, Jalesh N. Panicker, Jon Stone, Dani Coombe, Kianoush Nazarpour

**Affiliations:** 1 MoveR Lab, School of Informatics, The University of Edinburgh, Scotland, United Kingdom; 2 Royal Infirmary of Edinburgh, The University of Edinburgh, Scotland, United Kingdom; 3 Department of Urology, Victoria Hospital, National Health Service (NHS) Fife, Kirkcaldy, United Kingdom; 4 National Hospital for Neurology and Neurosurgery, University College London Hospital, London, United Kingdom; 5 Fowler’s Syndrome UK, London, United Kingdom

**Keywords:** case series, Fowler’s syndrome, neuromodulation, pilot study, TTNS

## Abstract

Fowler’s Syndrome is a critically under-researched area of Women’s Urological health. The condition, which causes severe urinary retention in the absence of any obvious obstructive pathology, lacks any non-invasive options for treatment. Transcutaneous Tibial Nerve Stimulation (TTNS) is an established treatment strategy for disorders of incontinence that is non-invasive, safe, and efficacious. Computer based modelling and preliminary animal experimentation indicates that ultra-low frequency TTNS (1–2 Hz) may induce an excitatory effect on bladder function that is of particular therapeutic relevance to Fowler’s Syndrome. Therefore, the present case series describes a three-armed randomised-control (crossover) pilot study conducted from a biomechanical perspective in a population suffering from Fowler’s Syndrome. The study aimed to address the question of if the novel application of low-frequency TTNS could ameliorate the symptoms of Fowler’s Syndrome. Patients (*N* = 6) were administered either ultra-low frequency TTNS (1 Hz) or placebo. The objective outcome measure of the study was the change in voiding efficiency in response to intervention. 50% (*N* = 3) participants displayed a positive response to treatment, with a median increase in Voiding Efficiency of 6.3 to 12.6% across test conditions. Two participants displayed a negative response to the intervention, (median −3.6 to −20.9%) which may have been due to placebo effects but should be considered in future. The study indicated that TTNS may be capable of ameliorating the symptoms of Fowler’s Syndrome, to a significant degree in one case. The work provides a foundation for future clinical research. The authors hope that should the results be confirmed by further clinical study they will have considerable impact on patient care.

## 1 Introduction

Despite being first described in 1985 by Fowler and Kirby (1), Fowler’s Syndrome remains a critically under-researched aspect of Women’s Urological health. The basic diagnostic criteria for the condition remain unformalised, but patients universally present with severe urinary retention associated with overactivity in the External Urethral Sphincter Osman and Chapple (2). Sacral Nerve Stimulation is the only effective treatment option available to patients Cox et al. (3). Though effective Swinn et al. (4), Sacral Nerve Stimulation requires surgery and possesses a high rate of complication Feloney et al. (5).

Transcutaneous Tibial Nerve Stimulation (TTNS) may possess the ability to ameliorate the symptoms of Fowler’s Syndrome. Preliminary evidence from animal models suggests that TTNS, which is normally inhibitory, may induce a rapid excitation of the bladder if applied at an altered dose (i.e., from the standard 10–20 Hz)Li et al. (6). Ultra-low (1–5 Hz) stimulation has been shown to induce an excitatory effect on bladder function in feline models Li et al. (7)Theisen et al. (8)Moazzam et al. (9). Additionally, previous work by the author has corroborated these findings via computer modelling, suggesting that an unexplored mechanism of action within the Periaqueductal Gray (PAG) may be the cause of this effect McConnell-Trevillion et al. (10).

The goal of this pilot series was to apply evidence from this biomechanical perspective and preliminarily explore if the excitatory effects of TTNS induce a clinically relevant change in a pathologically retentive population. All participants presented with a formal diagnosis of Fowler’s Syndrome, accompanied by severe urinary retention and lower-urinary-tract pain in all cases.

## 2 Materials and methods

**Figure 1:**
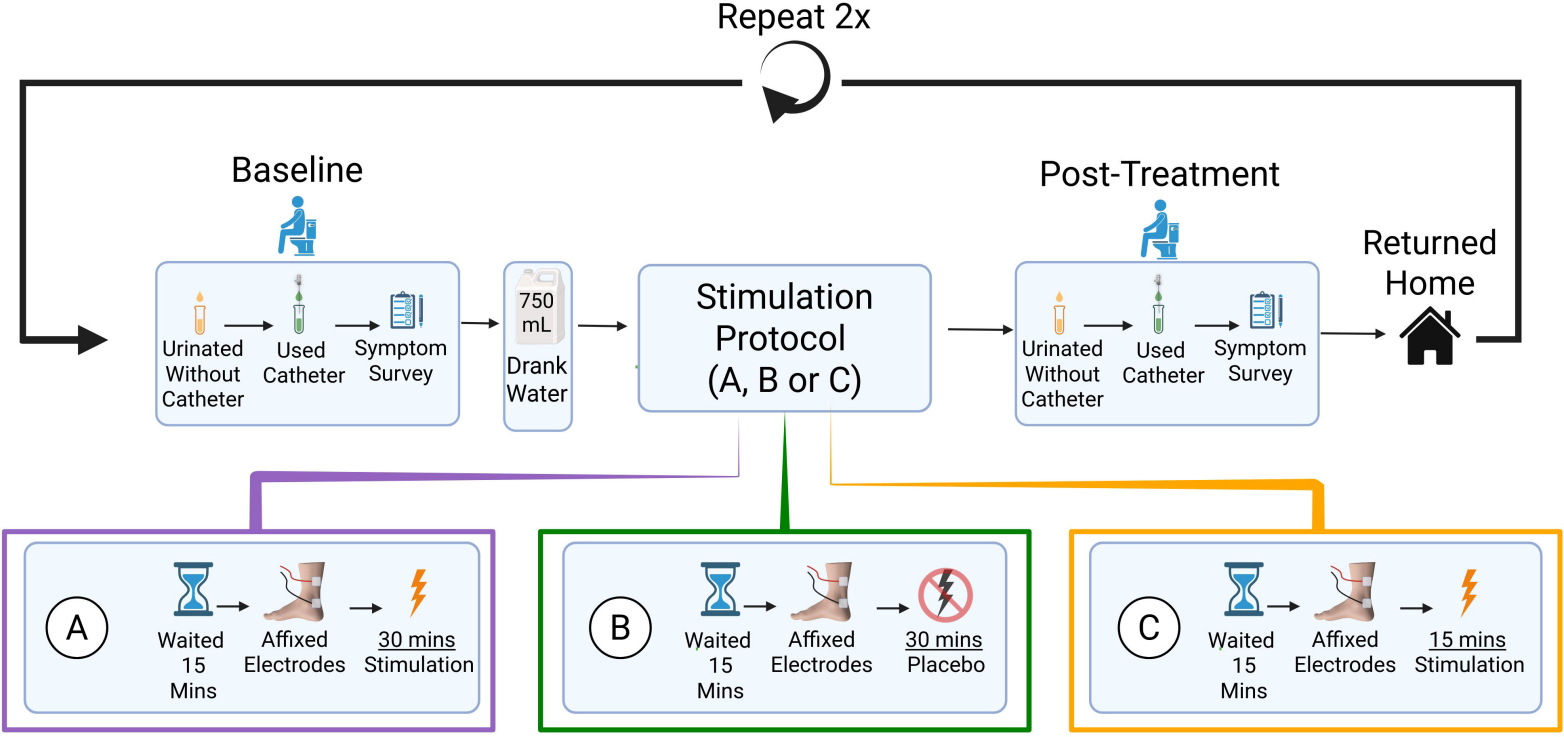
Graphical overview of pilot sudy mthodology. Participants underwent all conditions across three clinic visits **(A)** LEFT 30 Minutes at 1 Hz TTNS; **(B)** Middle: 30 Minutes of Placebo; **(C)** Right: 15 Minutes at 1 Hz TTNS). Order of condition allocation pseudorandomly determined across participants via custom python scripting which ensured equal distribution across the range of possible orders of experimental testing (randomisation ratio of 1:1:1, block size of 3).

A graphical representation of the experimental methodology is detailed in **Figure 1**. The study employed a placebo-controlled randomised three-armed crossover design, where participants were administered all three experimental interventions. Each intervention was administered at least 24 hours apart, ensuring all three conditions were completed within a three-week time frame. To establish an accurate baseline, before each session participants were asked to abstain from any alcohol, caffeine, or nicotine for 12-hours (as these substances have been shown to influence lower urinary tract function Burn et al. (11)Maughan and Griffin (12)Taari et al. (13)), and any fluids for 2-hours. Participants were not required to halt any ongoing treatment and could attend the experiment even if a supra-pubic catheter was fitted.

Participants attended three sessions, given the pilot nature of the study and a focus on immediate effects no follow up was conducted. In all cases they were asked to first empty their bladder as much as they were able without catheterising, and then to empty the remainder of their bladder with the catheterisation equipment they typically used. Participants were then asked to consume 750ml of water before being administered one of the following simulation protocols: low-frequency TTNS of 1 Hz for 30-minutes (condition A), placebo-stimulation for 30-minutes (condition B), or low-frequency TTNS for 15-minutes (condition C). Upon completion of the stimulation protocol, participants were then asked to repeat the assisted/unassisted urination procedure conducted during baseline establishment to ascertain any changes that arose as a result of TTNS administration.

Where TTNS was administered, it was conducted in line with standard guidance Bhide et al. (14): Hydrogel electrodes were placed 1 cm posterior (cathode) and 10 cm cephalad (anode) to the medial malleolus. Stimulation intensity was adjusted according to each participant such that it was sufficient to reach at motor threshold (where a fanning or twitching of the toes was observed), with a pulse-width of 200 *µs*, and stimulation frequency of 1 Hz. Where a placebo was applied, the same procedure was followed with the exception of electrical activity where no current was passed to the electrodes. Participants were not made aware of the presence of a placebo condition, nor which intervention was applied at each session. Additionally, participants were informed that it was entirely normal not to feel any sensation at all in some cases. A full equipment list may be found attached in the **Supplementary Materials**.

The primary objective outcome measure of the study was the change in *voiding efficiency* (VE) between baseline and post-intervention (Δ%*V E*). Given the reliance of the population on catheterisation to urinate, VE was here defined as the definition of VE was specified by the relationship shown in **Equation 1**.

(1) $$\%VE=\frac{Vol_{unassisted}}{Vol_{unassisted}+Vol_{catheterised}}\times 100$$

## 3 Results

A total of *N* = 8 adult women (*>* 18 Y.O.) with a formal diagnosis of Fowler’s Syndrome were recruited to take part in the study. A total of *N* = 6 participants completed the full experiment (mean age: 50; range: 25—68). Of the two participants that dropped out, both did so for personal reasons unrelated to the study. All participants displayed the expected toe-flexion/motor response characteristic of correct electrode placement during TTNS. Additionally, the supplied current was well tolerated by all participants with no complaints of pain reported across experimental conditions.

**Figure 2:**
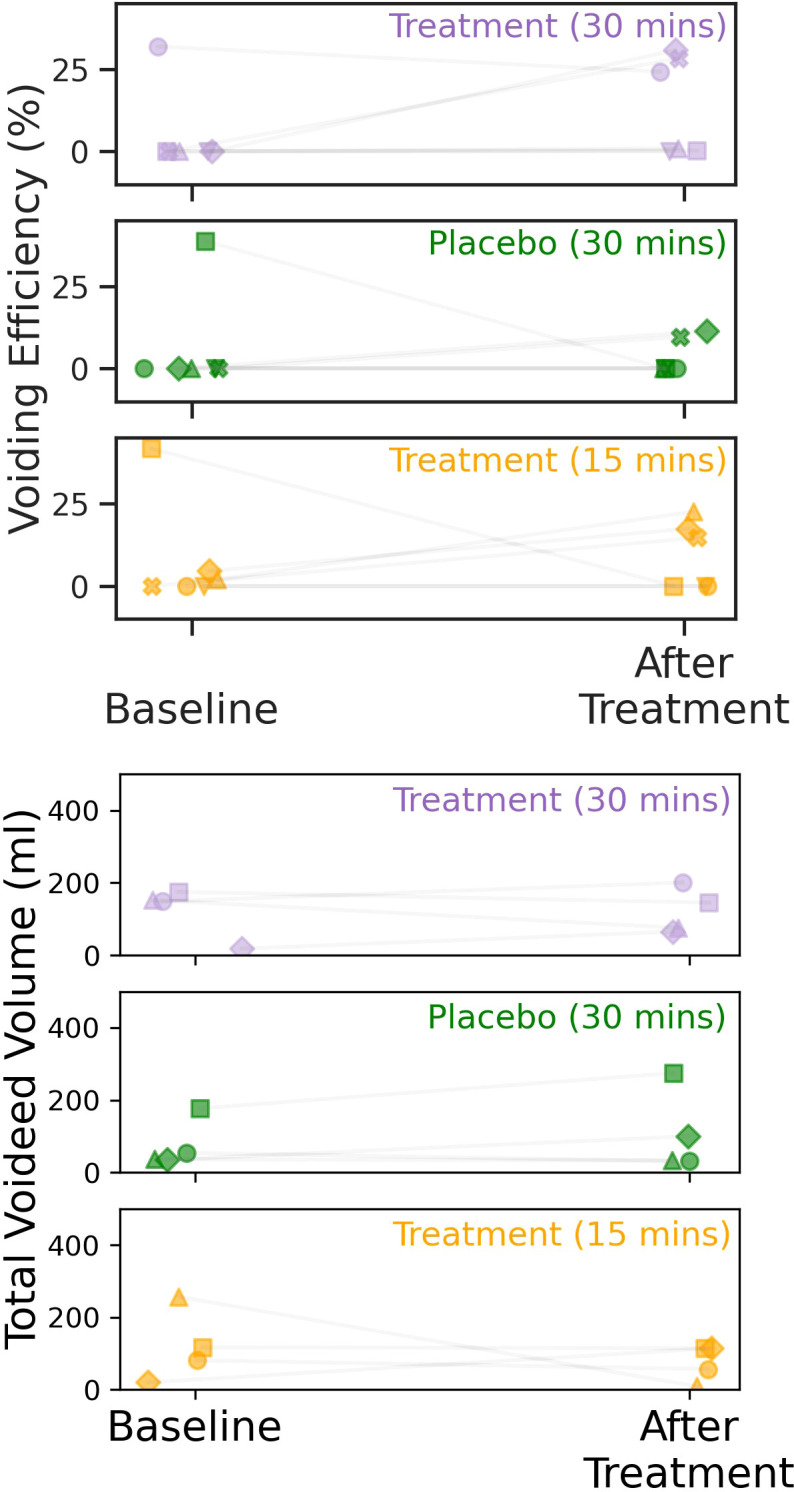
Change in voiding efficiency (top) and total voided volume (bottom) in response to TTNS or placebo intervention. Shown are all participants regardless of response to intervention (*N* = 6), represented by unique markers.

Of the six participants that completed all three experimental sessions, 50% displayed a positive change in voiding-efficiency in response to intervention (see **Figure 2**, top). In other words, 50% of the participants were able to void a greater proportion of the bladder without the assistance of a catheter after any form of intervention. The average magnitude of the change in efficiency across all participants (responsive, and non-responsive) was greater in the test conditions (median Δ%*V E*_30_*_m_*= 12.6, Δ%*V E*_15_*_m_*= 6.3) than in response to placebo (median Δ%*V E_placebo_*= 0.0). Analysing individual responses, magnitude changes were varied. In the test conditions two of the positive responders displayed similar changes to VE, while the last displayed little to no change in the 30-minute but larger shift in the 15-minute condition respectively: (Δ%*V E*_30_*_m_*= 30.8, 28.3, and 0.94; Δ%*V E*_15_*_m_*= 12.9, 14.6, and 20.6 respectively). Conversely, in the placebo condition, magnitude changes were smaller across the board (Δ%*V E_placebo_*= 11.5, 9.7, 0.0).

Analysing the total voided volume revealed that there was little positive change in total voided-volume across both test (median Δ*TotV ol*_30_*_m_*= −3.8*ml*, Δ*TotV ol*_15_*_m_*= −32.4*ml*) and placebo conditions (Δ*TotV ol*_15_*_m_*= 19.8*ml*, see **Figure 2**, bottom).

Two participants displayed a negative change in voiding efficiency in response to intervention. The first of the two participants displayed this effect in both the placebo and test (15-minute TTNS) conditions but not in the 30-minute condition (Δ%*V E_placebo_*= -38.8; Δ%*V E*_15_*_m_*= -41.8; Δ%*V E*_30_*_m_*= 0.3), while the latter displayed the change in only one of the three sessions (Δ%*V E_placebo_* = 0.0;Δ%*V E*_15_*_m_*= 0.0; Δ%*V E*_30_*_m_*= -7.7).

Finally, one participant displayed a considerable positive response to TTNS. They reported that they had been in complete retention for 15 years at the time of the study. However, after TTNS (but not placebo), they reported the ability to pass a small volume of urine unassisted (Δ%*V E* ≈2%).

## 4 Discussion

This work lays the foundation for critical large-scale clinical work and serves as both basic biomechanical experimental research, and a case-series showcasing the novel application of an approved intervention. Consequently this research is beneficial to both scientists and healthcare professionals involved in the treatment of urological pathology.

The findings are promising for several reasons. Firstly, while the intervention allowed for the voiding of only several millilitres of urine, the fact that low-frequency TTNS (but not placebo) appeared to ameliorate some of the effects of long-term retention is of particular therapeutic importance. Secondly, 50% of participants reported an increase in urination ease post-TTNS, particularly after the 30-minute treatment regimen where Δ%*V E* was greatest, with a similar magnitude of positive change noted in two of the three positive respondents. This increased ease was because patients were able to pass more urine without catheterisation after treatment, as the VE measure took into account changes in relative unassisted:assisted (catheterised) volume. Moreover, one participant in fact urinated *less* in total yet displayed a positive increase in VE). Nevertheless, this a minor increase in total voided volume was observed and as such should be kept in mind when interpreting the results.

Finally, two participants did display a negative response to the intervention however one participant had a negative response to all interventions suggesting a no-cebo response rather than a TTNS specific effect. This being said, the remaining negative-responder displayed the a deterioration after solely the longer TTNS session, but not placebo, indicating that there may be a potential for the intervention to induce a negative effect on voiding efficiency. To this end, while it cannot be said at this time why this deterioration may have occurred, as the results point to this adverse effect as a potential outcome any future clinical trials must include a close monitoring of participants for any signs of deterioration.

The present work arose as a result of basic biomechanical research McConnell-Trevillion et al. (10) which proposed a hypothetical framework, according to which TTNS may induce a differential effect through an alteration of supraspinal afferent processing. These results further support this hypothetical mechanism of action by demonstrating that the putative excitatory effect generalises to a population suffering from pathological urinary retention. Outwith this basic scientific benefit, the case series is promising to urological healthcare as it indicates that an established NHS intervention for treating LUT dysfunction NHS Torbay and South Devon Foundation Trust (15), may be applied to novel circumstances with positive effect. Moreover, they demonstrate that this intervention could be of use in facilitating unassisted urination in cases where patients require Intermittent Self Catheterisation but struggle due to pain or muscle spasm. Finally, they provide insight into a critically under-explored aspect of TTNS: its dose-related efficacy.

This insight is particularly important as, historically, TTNS has been administered according to a rough set of standardised parameters (10–20 Hz, 200*µs* pulse width, at or near motor threshold) Booth et al. (16). However, the scientific evidence supporting the use of these parameters is limited. The original publication detailing Stoller Afferent Nerve Stimulation (SANS) provided little information regarding the optimal parameters or their rationale Stoller et al. (17). Moreover, in spite of initial mentions of TTNS being administered at lower-frequencies (*<*10 Hz) Mcguire et al. (18), all subsequent work in the field utilised the “standard” 20Hz/200 *µs* parameters as clinical truth. As a consequence of assuming these parameters to be optimal, little work has been conducted concerning the dose-related efficacy of the intervention. To this end, the present results provide critical justification for further study of the frequency-dependent effects of TTNS; not only on the basis of therapeutic benefits to Fowler’s patients but also to firmly establish the scientific baseline of the intervention. Further work on duration (15 vs 30-minute vs other durations) of treatment is also required. On the topic of this final point, there exists a critical limitation within both contemporary literature, and patient treatment. Specifically, there is little standardisation of other TTNS parameters across the literature Shang et al. (19). Namely, the administration duration, and frequency (i.e., the number of times TTNS is applied per week/month, rather than the electrical frequency of the intervention). Prior work has indicated this is a topic of particular interest, as modification of the number of sessions per week Ergin et al. (20), and the total duration of the course of treatment Cheng et al. (21) can have a significant effect on patient outcomes, in cases of incontinence. To this end, further work would be well placed to explore other optimal stimulation parameters, as these facets of TTNS remain critically under-explored.

### 4.1 Limitations

Though these results are preliminarily promising, there remain several limitations to the work that must be addressed. Firstly, the sample size of the case series prevents major generalisation of these results and limits the conclusions that may be drawn from the findings. Nevertheless, they are promising and justify further analysis via large-scale study. Additionally, the issue of fluid restriction remains problematic. Namely, that fluid restriction may have contributed to the relatively low voiding efficiencies observed in the test population and that doing so may have obfuscated true bladder baseline. Though a two-hour fluid restriction was imposed to attempt to normalise bladder baselines across participants, it should be noted that this may not represent a baseline, and may have contributed to relatively low voiding efficiencies observed across the population This being said, despite this limitation, there was still an observed effect in the responsive subpopulation that was of a greater magnitude than placebo, moreover the same fluid restriction process was applied to all participants. As such it is expected that any potential reduction in voiding efficiency due to fluid restriction would be present across all conditions and therefore accounted for in *post-hoc* analysis. Future work could remedy this limitation by instead conducting baseline measurement in the absence of fluid restriction, immediately after ingestion of the test water volume. Finally, these results must be considered through the lens of the potentially limited placebo blinding. Given the nature of the intervention (with TTNS causing a distinct sensation and motor response) it was difficult to fully blind participants during the placebo condition, as they were aware that some difference in stimulation was present. As mentioned, actions were taken to attempt to mitigate this, with participants being shielded from the presence of a placebo condition, and being informed that several different types of stimulation would be applied as part of the study and that it was entirely normal not to feel any sensation at all in some cases. Nevertheless, it cannot be said that there was no impact of the awareness of a differently applied stimulation paradigm. Future work should attempt to remedy this, potentially via application of a sham stimulus known to have no effect on bladder function (e.g., other foot stimulation, or stimulation of a different region of the lower body).

Future work should address these limitations and should place a greater focus on recruitment of a homogeneous patient group via the careful documentation of patient diagnostic criteria (e.g., High UPP, normal detrusor activity and a regular uninhibited use of CISC). Moreover, changes to the methodology will increase the accuracy of the objective outcome measures (e.g., the use of ultrasonic bladder scanners to determine pre-void bladder state, or uroflowmetry to measure critical physiological parameters) and ensure a consistent baseline of participant hydration. Nevertheless, despite these issues this research demonstrates a preliminary effect of low-frequency TTNS on bladder function that may be relevant to those suffering from non-obstructive disorders of retention.

## 5 Conclusion

The authors do not wish to overemphasise the results of the present study, as they were a pilot case series and therefore do not provide definitive proof of an excitatory effect. Nevertheless, we believe that the present experimental evidence may have a significant impact on patient care, should the results be confirmed by further clinical study. Should these findings bear fruit, we believe that something as simple as a small modification to an established technique could have significant impacts on outcomes in a critically under-represented patient population.

## Data Availability

The original contributions presented in the study are included in the article/**Supplementary Material**. Further inquiries can be directed to the corresponding author.

## References

[1] Fowler CJ Kirby RS . Abnormal electromyographic activity (decelerating burst and complex repetitive discharges) in the striated muscle of the urethral sphincter in 5 women with persisting urinary retention. Br J Urol. (1985) 57:67–70. doi: 10.1111/J.1464-410X.1985.TB08988.X. PMID: 4038618

[2] Osman NI Chapple CR . Fowler’s syndrome — a cause of unexplained urinary retention in young women? Nat Rev Urol. (2013) 11:87–98. doi: 10.1038/nrurol.2013.277. PMID: 24323131

[3] Cox ER Panicker JN Coombe D Selai C Ellis D Stone J . Fowler’s syndrome — patient led phenotyping of 265 patients. Continence. (2024) 12:101710. doi: 10.1016/j.cont.2024.101710. PMID: 38826717

[4] Swinn MJ Kitchen ND Goodwin RJ Fowler CJ . Sacral neuromodulation for women with Fowler’s syndrome. Eur Urol. (2000) 38:439–43. doi: 10.1159/000020321. PMID: 11025383

[5] Feloney MP Stauss K Leslie SW . Sacral neuromodulation. In: StatPearls. Treasure Island (FL): StatPearls Publishing. (2024). 33620828

[6] Li X Li X Liao L . Mechanism of action of tibial nerve stimulation in the treatment of lower urinary tract dysfunction. Neuromodul.: Technol At. Neural Interface. (2023) 27(2):256–266. doi: 10.1016/J.NEUROM.2023.03.017. PMID: 37178068

[7] Li S Browning J Theisen K Yecies T Shen B Wang J . Prolonged nonobstructive urinary retention induced by tibial nerve stimulation in cats. Am J Physiol Regul Intgr. Comp Physiol. (2020) 318:R428–34. doi: 10.1152/ajpregu.00277.2019 PMC7052595 31913685

[8] Theisen K Browning J Li X Li S Shen B Wang J . Frequency dependent tibial neuromodulation of bladder underactivity and overactivity in cats. Neuromodul.: Technol At. Neural Interface. (2018) 21:700–6. doi: 10.1111/NER.12792. PMID: 29949663 PMC6175618

[9] Moazzam Z Duke AR Yoo PB . Inhibition and excitation of bladder function by tibial nerve stimulation using a wirelessly powered implant: an acute study in anesthetized cats. J Urol. (2016) 196:926–33. doi: 10.1016/J.JURO.2016.04.077. PMID: 27154823

[10] McConnell-Trevillion A Jabbari M Ju W Lister E Erfanian A Mitra S . Low frequency tibial neuromodulation increases voiding activity - a human pilot study and computational model. eLife. (2025) 14. doi: 10.7554/ELIFE.106174.1 PMC13544907 42695443

[11] Burn JH Truelove LH And BA Burn I . The antidiuretic action of nicotine and of smoking. (1946) 17(33):1026–1031. doi: 10.1136/bmj.1.4394.403 20988903

[12] Maughan RJ Griffin J . Caffeine ingestion and fluid balance: a review. J Hum Nutr Diet. (2003) 16:411–20. doi: 10.1046/J.1365-277X.2003.00477.X. PMID: 19774754

[13] Taari K Ruutu M Lehtonen T . Effect of alcohol on bladder function: a uroflowmetric and cystometric study. Neurourol. Urodynamics. (1990) 9:591–4. doi: 10.1002/NAU.1930090604. PMID: 41531421

[14] Bhide AA Tailor V Fernando R Khullar V Digesu GA . Posterior tibial nerve stimulation for overactive bladder — techniques and efficacy. Int Urogynecol. J. (2020) 31:865. doi: 10.1007/S00192-019-04186-3. PMID: 31853597 PMC7210232

[15] NHS Torbay and South Devon Foundation Trust . Transcutaneous tibial nerve stimulation for overactive bladder physiotherapy advice. (2024).

[16] Booth J Connelly L Dickson S Duncan F Lawrence M . The effectiveness of transcutaneous tibial nerve stimulation (TTNS) for adults with overactive bladder syndrome: a systematic review. Neurourol. Urodynamics. (2018) 37:528–41. doi: 10.1002/NAU.23351. PMID: 28731583

[17] Stoller M Copeland S Millard R Murnaghan G . The efficacy of acupuncture in reversing the unstable bladder in pig-tailed monkeys. J Urol. (1987) 137:104a. doi: 10.1016/S0022-5347(17)75152-X

[18] Mcguire EJ Shi-Chun Z Horwinski ER Lytton B . Treatment of motor and sensory detrusor instability by electrical stimulation. J Urol. (1983) 129:78–9. doi: 10.1016/S0022-5347(17)51928-X. PMID: 6600794

[19] Shang D Deng H Li C Wang Z Jin L Li X . Tibial nerve stimulation for overactive bladder: a literature review of stimulation parameters. Trans Androl. Urol. (2026) 15:67. doi: 10.21037/tau-2025-aw-774. PMID: 41809796 PMC12968878

[20] Ergin IE Asdemir A Saygın H Korgalı E . Impact of weekly application frequency on the efficacy of tibial nerve stimulation therapy in the treatment of overactive bladder. Int Urogynecol. J. (2025) 37:87–92. doi: 10.1007/S00192-025-06217-8/METRICS 40668392

[21] Cheng F Wang J Wang D Wang P Zhang Y Song G . Extended-course transcutaneous tibial nerve stimulation for pediatric overactive bladder: a 6-month prospective single-arm study. World J Urol. (2026) 44:218. doi: 10.1007/S00345-026-06249-9. PMID: 41779059 PMC12960355

