# Supplementary material for: Case Report: Low-frequency tibial nerve stimulation: demonstrating a novel therapeutic option for Fowler’s syndrome through a pilot case series
Source: Front Urol. 2026 May 20;6:1854503. doi: 10.3389/fruro.2026.1854503 (PMC13229719; doi:10.3389/fruro.2026.1854503)
Supplement: Supplementary file 1 [file DataSheet1.pdf]

Exclusion Criteria

**Exclusion Criteria**
Those younger than 18 years of age
Those with any active urinary tract infection (UTI)
Those with pre-existing cardiovascular conditions
Those with existing implanted treatment devices actively in use at the time of the study (pacemakers, neuromodulators, defibrillators, etc.)
Those with uncontrolled epilepsy
Those with broken/fragile/thin/infected skin around the treatment site (ankle)
Pregnant individuals or individuals planning pregnancy during the study
Those with severe nerve damage or spinal cord injury
Those with a lack of capacity to provide consent
Those with previous surgical intervention for the treatment of IUR/FS (or otherwise) that considerably alters the anatomy of the lower-urinary tract

Supplementary Table 1: Study Participant Exclusion Criteria

Detailed Protocol and Equipment List

[redacted]

This document contains guidance for researchers carrying out the Urinary Retention Tibial Nerve Stimulation Validation study [redacted] Information may be viewed in more detail in the supplementary materials if required. If you have any questions or issues, please contact either [redacted] or [redacted]

[redacted]

Table of Contents

Pre-Trial Preparation ____ 3

Running the Trial ____ 4

- Study Equipment Checklist ____ 4
- Pre-Study ____ 5
- Stimulation Experiment – Baseline Establishment ____ 5
- Stimulation Experiment – Intervention ____ 6
- Stimulation Experiment - Neurostimulation ____ 8
- Post-Study ____ 10

Appendix A – Template Email for Participants (After Consent is Obtained) ____ 12

Appendix B – Participant ID to Order Sheet. ____ 13

[redacted]

Pre-Trial Preparation

Before **any research is conducted,** full informed consent must be obtained from prospective participants. After obtaining this, using the participants preferences and suggested times, organise a time and place that suits them best.

1. Send interested participants the **participant information sheet** via email.
2. Making sure to answer any of their questions or concerns, ensure the form is fully completed!
3. Once participants have completed the form, Create an ID entry in the participant ID sheet stored on the lab AFS server: [redacted]
4. Once participants have completed the form, **using this ID number** enter their demographic information anddpreferences into a template copy (labelled with their ID number) in the [redacted]

Once consent has been obtained, you can **arrange a time and date that suits participants.**

1. Look over the shared calendar and find a free slot (**THAT CAN BE BOOKED OFF THROUGH** [redacted]
    a. IF A CHAPERONE IS REQUIRED, FIND IF THEY ARE FREE DURING THIS TIME AND ARRANGE THIS WITH THEM.
    b. Ensure there is no overlap between participants (e.g., if they wish to wait at the centre during the water restriction period).
2. Create a calendar event in RSPACE and circulate this to **both the participant, and the chaperone if required.**
3. Send the participant a confirmation email with instructions on what to do before the session (abstain from caffeine and cigarettes for 12 hours, abstain from any fluids for 2 hours). (**see appendix A for a template email**).

[redacted]

Running the Trial

To ensure scientific validity, when the participant has arrived, the following methodology should be carried out in the same manner every time. Shown below is a graphical representation of the experimental methodology for rapid reference if required. Note, A, B, and C are to be undertaken on **different days at least 24 hours apart.**

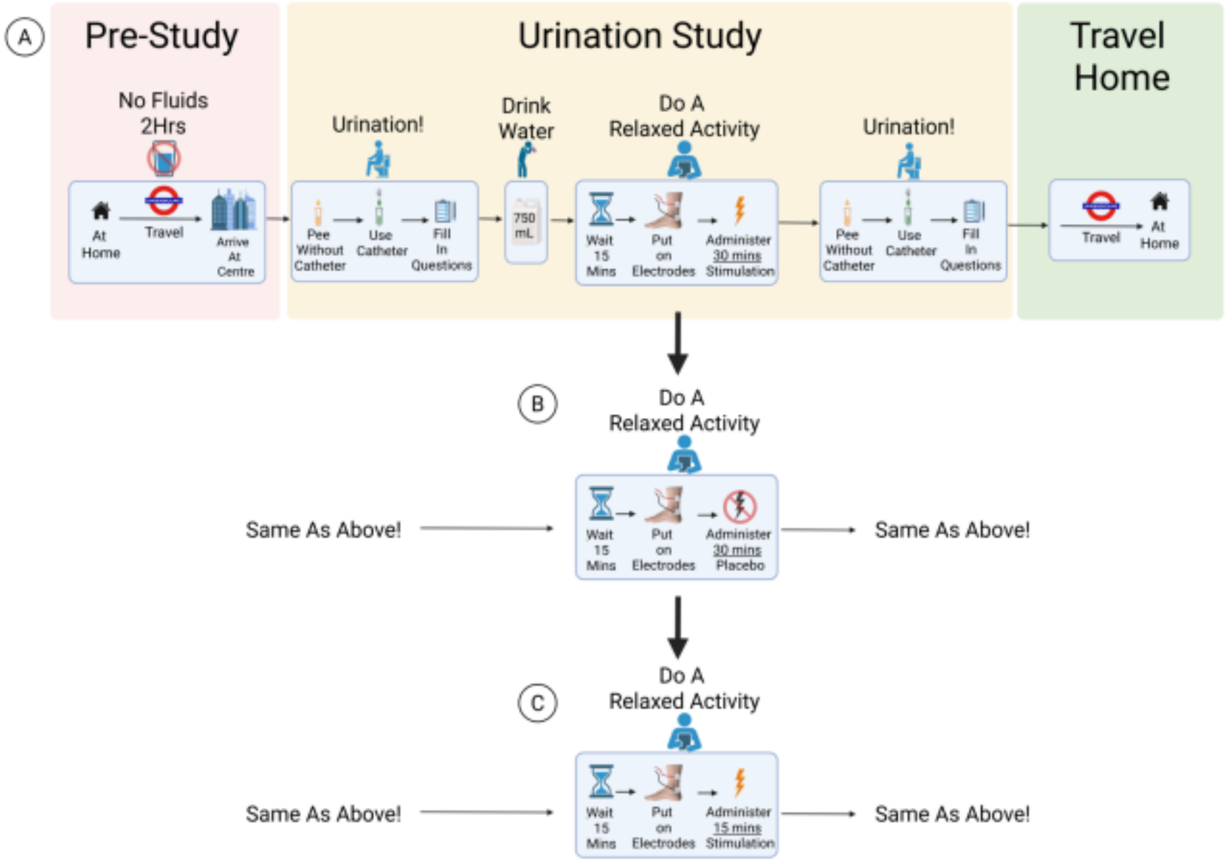

S. Figure 1 Graphical overview of the planned study. Note that participants will undergo conditions A, B, and C **in a randomised order.**

Study Equipment Checklist

Stimulator	
Electrode Packs (Nx for participants)	
Cables (electrode, power cables,	

[redacted]

stimulator controller, usb cable)	
Bedpans (x4 per participant)	
Water (1x 750ml bottle per participant)	
Cups (1x per participant)	
Scales	
Disinfectant	
Cotton Balls	
Catheters (2x per participant if required)	

Pre-Study

If participants wish to wait out the water restriction at the centre, ensure additional time is allocated beforehand (2 hours). Welcome the participant to the study, make them feel relaxed and at ease, andwer any questions they might have (they may have some new ones after some time to think). Once they are settled, carry out the following:

1. Open the participant's RSPACE documentation for rapid entry
2. Introduce yourself and inform them that the study will be done the same way they have been made aware of,
3. **Confirm with participants that they have abstained from caffeine/nicotine for 12 hours, and any fluids for 2 hours.**
4. Ask if they have any concerns or things that I should be aware of before we begin.
5. Note this in the RSPACE form.
6. **WEIGH THE BEDPANS FOR BEFORE/AFTER WEIGHTS AND NOTE THEM.**

Stimulation Experiment – Baseline Establishment

Begin the study by informing participants that we're hoping to establish a baseline level before we administer the stimulation.

1. Inform them that you are going to ask them to visit the toilet (will show them where this is). And do three things:

[redacted]

a. Urinate as much as they can without a catheter into the bedpan labelled NO CATHETER. (State that we will put this in the front of the toilet).
b. Set this aside, insert the bedpan labelled CATHETER and then as they would normally catheterise and empty their bladder the rest of the way into this bedpan.
c. **NOTE: Let them know that if required I can change these around for them.**
d. Come back to the room, and then fill out an online form about how they found this toilet visit.

2. Allow participants to carry this out. Show them where the toilet is, and ask if they would like any assistance. Tell them they may take as long as they need to do this.
3. Once participants are finished, escort them back to the room with chaperone and then take measurements.
    a. Wearing **appropriate PPE,** PLACE THE SCALES ON STABLE SURFACE AND TARE THEM
    b. PLACE EACH FILLED BEDPAN ON THE SCALES, AND NOTE THE WEIGHT IN GRAMS.
    c. EMPTY THE BEDPANS INTO THE TOILET AND THEN APPROPRIATELY DISPOSE OF THEM.
4. Ask participants to fill in the online form (provide a QR code for them).

Stimulation Experiment – Intervention

1. Inform participants that I would now like them to drink some water. They will have to drink 750ml of water. Ensure they know they can take as long as required to do so, and that they have a range of options available (cold, warm etc.).
2. Allow participants to drink the water and then during the digestion period explain that the next stage of the study will be to administer the intervention.
3. Look at the ID order document (appendix B) and note the appropriate intervention:
    a. A) 30mins @ 1Hz
    b. B) 30mins @ 0Hz
    c. C) 15mins @ 1Hz
4. Inform the participant that you are going to prepare their skin for the second part of the experimental study.
    a. Disinfect the skin gently using cotton balls soaked in isopropyl alcohol (70% by volume).

[redacted]

b. Affix electrodes to the skin (**without attaching them to the stimulator**) 1cm posterior /10cm cephalad to the medial malleolus (see fig. 2).

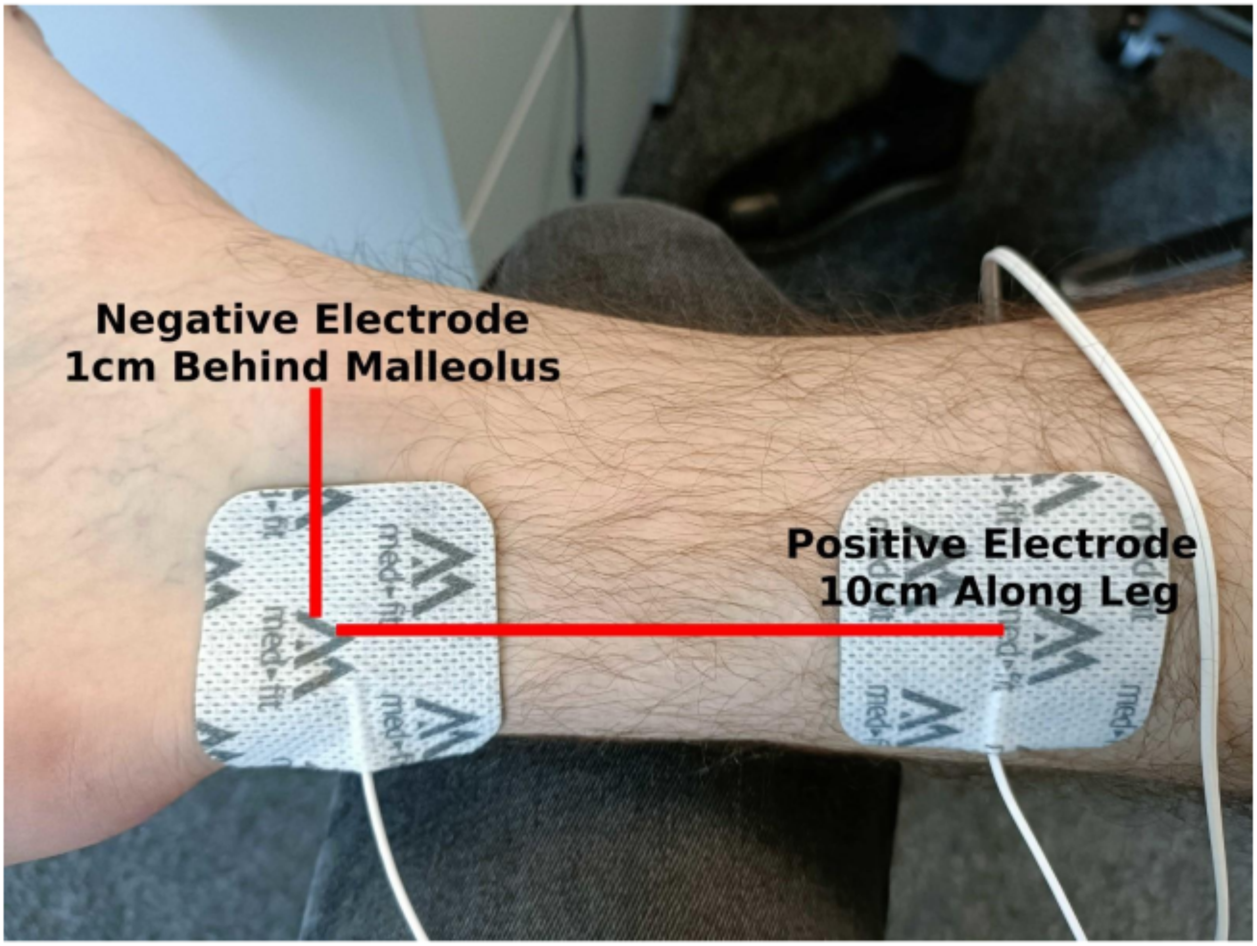

S. Figure 2 **Position of electrodes relative to Medial Malleolus.** Placement should be measured from the centre of each electrode relative to the centre of the medial malleolus (note: 1cm is approximately 1 finger width).

[redacted]

Stimulation Experiment - Neurostimulation

1. Inform participants that we will now begin administering a gentle stimulation of their ankle. Inform them that this may cause some discomfort but will not be painful. If they have visited before, note that we are utilising several different interventions and that this one may feel slightly different than what they are used to.
2. **Remember to inform participants that they may halt the experiment at any time and for any reason.**
3. Attach the electrode pads to the DS7A, positive lead to the electrode nearer the knee, negative lead to the electrode nearer to the foot.
4. Connect to the DS7 control unit
    a. Using the bluetooth app (STBLESensor Classic) connect to [redacted]
    b. **If the device is not listed press Reset SM4.**
5. Begin stimulation protocol in accordance with the group the participant has been allocated to. **Ensure DS7 is correctly setup (see fig. 3)**
    a. For 1Hz stimulation, press the bulb symbol **once**
    [redacted] b. For placebo stimulation, leave the top right switch on the DS7A in the off position. Inform participants in this group they may not feel anything at all, but turn up the intensity knob to 300 to maintain the illusion of stimulation.
6. Increase the intensity of the stimulation to motor threshold (where toe flexion or fanning is observed) by turning the intensity knob slowly. (see fig. 3).
7. **At the same time, start a timer.**
8. After the appropriate amount of time has passed halt stimulation.
    a. A) 30 Minutes
    b. B) 30 Minutes
    c. C) 15 Minutes

[redacted]

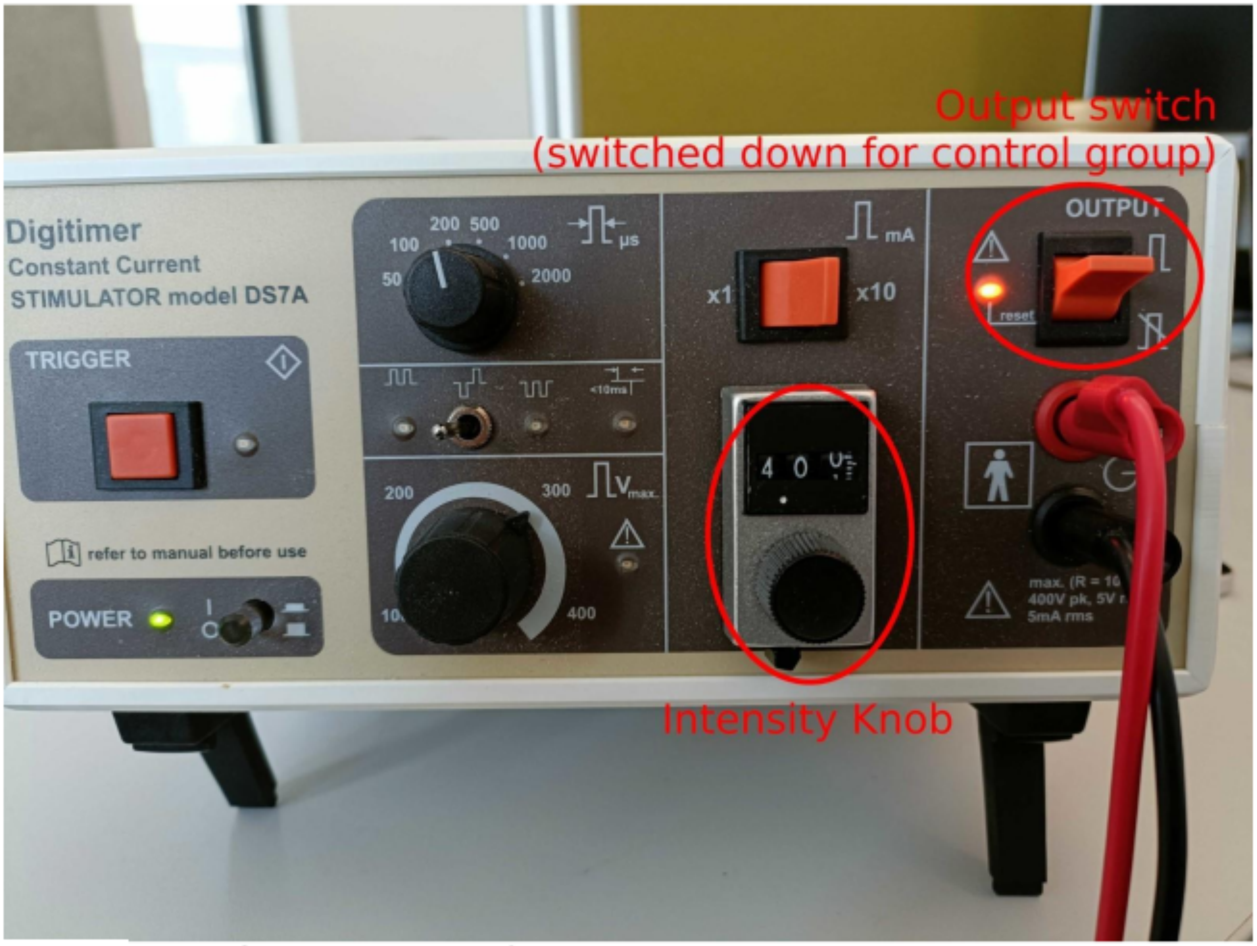

S. Figure 3**. The DS7A and settings for stimulation.** Device should be setup as pictured. In the case of the control group (group B) the output switch should be in the down position. Current intensity is controlled via the intensity knob.

At this point, inform participants that they will now be asked to do exactly the same thing that they did when they first arrived. AS BEFORE:

1. Inform them that you are going to ask them to visit the toilet (will show them where this is). And do three things:
    a. Urinate as much as they can without a catheter into the bedpan labelled NO CATHETER. (State that we will put this in the front of the toilet).

[redacted]

   b. Set this aside, insert the bedpan labelled CATHETER and then as they would normally catheterise and empty their bladder the rest of the way into this bedpan.
   c. **NOTE: Let them know that if required I can change these around for them.**
   d. Come back to the room, and then fill out an online form about how they found this toilet visit.
2. Allow participants to carry this out. Show them where the toilet is, and ask if they would like any assistance. Tell them they may take as long as they need to do this.
3. Once participants are finished, escort them back to the room with chaperone and then take measurements.
   a. Wearing **appropriate PPE,** PLACE THE SCALES ON STABLE SURFACE AND TARE THEM
   b. PLACE EACH FILLED BEDPAN ON THE SCALES, AND NOTE THE WEIGHT IN GRAMS.
   c. EMPTY THE BEDPANS INTO THE TOILET AND THEN APPROPRIATELY DISPOSE OF THEM.
4. Ask participants to fill in the online form (provide a QR code for them).

Post-Study

Inform participants that this part of the study is now complete.

1. Carefully remove the electrodes if participants are comfortable (or allow them to do this themselves).
2. Ensure participants are comfortable and not suffering any side-effects. Inform them that if they do, they are to inform me and if required visit a medical practicioner.

Remind them that they will be required to visit two more times if this is their fist visit.

- Arrange an appropriate time for their follow-up visit with them at this point. Note this down and like before **create an appropriate calendar entry in RSPACE to circulate to both them and the chaperone.**

After the participant has left, thank the chaperone and begin prep for the next session:

[redacted]

1. Lay out new bedpans and PPE equipment
2. Lay out fresh electrodes
3. Clean and disinfect any surfaces and the bathroom
4. Dispose of any waste or biohazardous materials appropriately.
5. Restock water if required
6. Export their qualtrics responses
7. Enter the results of the measurement into the participant's RSPACE file
8. Prepare the next participant's RSPACE entry for smooth transition

[redacted]

Appendix A – Template Email for Participants (After Consent is Obtained)

Dear [Participant],

Thank you for your interest in our Urinary Retention study and for completing our consent form.

This email provides more information about what's involved in the research and includes some available timeslots for your first session at our centre.

As a reminder, if you choose to take part in the study, we ask that you commit to attending [redacted]

During less active periods of the study, you'll be free to work remotely or conduct any activities that can be done while seated. While we are unable to reimburse you for your participation, we've designed the study to minimize the impact on your ongoing work as much as possible.

We currently have free timeslots on:

Please let me know which of these slots you would be able to attend, and I will get you booked in. If none of these times work for you, please don't hesitate to let me know, and I'll be happy to arrange an alternative timeslot.

Due to the high number of people who have expressed interest, slots are limited and are filling up quickly. We recommend letting us know your availability as soon as possible to secure your preferred time.

Before Your Session:

To ensure that our experiment is accurate and runs as smoothly as possible, please:

- **Avoid any nicotine/caffeine for 12 hours before your session.**
- **Avoid drinking any fluids for 2 hours before your session.**
- If you indicated you wish to use your own catheters, **please bring enough for at least 2 toilet visits with you to the session.**

[redacted]

- Please arrive at the centre on time for your slot. If you cannot attend for any reason, please let me know as soon as possible so we can offer the slot to someone else.

Getting To the Centre:

[redacted]

Thank you again for your interest in our study. If you have any questions at all, please don't hesitate to ask, and I'll be happy to answer them :)

I look forward to welcoming you to our centre!

Best Wishes,

Appendix B – Participant ID to Order Sheet.

ACB	CAB
P2	P1
P3	P4
P6	P5
P7	P8
P10	P9
P12	P11
P13	P14

[redacted]

P16	P15
P17	P18
P20	P19
P21	P22
P24	P23
P25	P26
P27	P28
P29	P30
P31	P32
P33	P34
P36	P35
P38	P37
P40	P39
P42	P41
P43	P44
P46	P45
P48	P47
P50	P49

Anonymised Raw Data

Supplementary Table 2: Quantitative Results - Symptom Severity. FU: Flare Up, BL_SP: Bladder Spasms, BL_Inflam: Bladder Inflammation, UR_Inflam: Urethral Inflammation

Participant ID	Condition	Symptom_Severity	Retention_FU	Pain_FU	Bl_Sp_FU	Bl_Inflam_FU	Ur_Inflam_FU
1	1hz_15	0	N	N	N	N	N
1	1hz_30	35	N	N	N	N	N
1	placebo	16	N	Y	Y	N	N
2	1hz_30	50	N	N	N	N	N
2	1hz_15	25	N	N	N	N	N
2	placebo	50	N	N	N	N	N
3	1hz_30	50	N	N	N	N	N
3	1hz_15	50	N	N	N	N	N
3	placebo	50	N	N	N	N	N
6	1hz_30	55	N	N	N	Y	N
6	placebo	38	N	N	N	N	Y
6	1hz_15	40	N	N	Y	Y	Y
7	1hz_30	0	N	N	N	N	N
7	1hz_15	0	N	N	N	N	N
7	placebo	0	N	N	N	N	N
8	1hz_15	25	N	N	N	N	N
8	placebo	41	N	N	N	N	N
8	1hz_30	51	N	N	N	N	N

Supplementary Table 3: Quantitative Results - Objective Outcome Measures

Participant ID	Baseline_Urination (ml)	Baseline_Cath (ml)	Baseline_VE (%)	Post_Treat_Urination (ml)	Post_Treat_Cath (ml)	Post_Treat_VE (%)	Delta_VE	Age
1	0.0	200.65	0.0	0.0	166.31	0.0	0.0	68
1	107.2	228.8	31.9047619047619	89.04	278.3	24.2391245167964	-7.66563738796547	68
1	0.0	102.58	0.0	0.0	147.98	0.0	0.0	68
2	0.0	93.97	0.0	0.149999999999999	53.62	0.278965966152127	0.278965966152127	52
2	123.61	172.4	41.75872436742	0.0	124.48	0.0	-41.75872436742	52
2	172.68	272.23	38.8123440695871	0.0	85.57	0.0	-38.8123440695871	52
3	0.0	148.65	0.0	1.9	201.16	0.935684034275583	0.935684034275583	60
3	1.61	81.24	1.94327097163549	16.57	56.85	22.568782348134	20.6255113764985	60
3	0.0	54.32	0.0	0.0	31.31	0.0	0.0	60
6	0.0	175.03	0.0	64.76	145.5	30.7999619518691	30.7999619518691	25
6	0.0	177.1	0.0	35.56	274.58	11.4657896433869	11.4657896433869	25
6	5.53	117.01	4.51281214297372	24.19	114.83	17.4003740468997	12.887561903926	25
7	0.0	152.73	0.0	0.0	75.98	0.0	0.0	63
7	0.0	256.96	0.0	0.0	9.37	0.0	0.0	63
7	0.0	36.52	0.0	0.0	32.9	0.0	0.0	63
8	0.0	20.16	0.0	19.61	114.38	14.6354205537727	14.6354205537727	34
8	0.0	35.61	0.0	10.73	100.16	9.67625574894039	9.67625574894039	34
8	0.0	18.47	0.0	25.69	65.11	28.2929515418502	28.2929515418502	34
